# Efficacy and Safety of Teneligliptin in Patients With Type 2 Diabetes Mellitus: A Systematic Review and Meta-Analysis of Randomized Controlled Trials

**DOI:** 10.3389/fphar.2018.00449

**Published:** 2018-05-04

**Authors:** Xiaoxuan Li, Xuefei Huang, Chongfei Bai, Dalian Qin, Shousong Cao, Qibing Mei, Yun Ye, Jianming Wu

**Affiliations:** ^1^Laboratory of Chinese Materia Medica, Department of Pharmacology, School of Pharmacy, Southwest Medical University, Luzhou, China; ^2^Department of Clinical Pharmacy, School of Pharmacy, Southwest Medical University, Luzhou, China; ^3^Department of Chinese Materia Medica, School of Pharmacy, Chengdu University of Traditional Chinese Medicine, Chengdu, China; ^4^Laboratory of Cancer Pharmacology, Department of Pharmacology, School of Pharmacy, Southwest Medical University, Luzhou, China; ^5^Department of Pharmacy, Affiliated Hospital of Southwest Medical University, Luzhou, China

**Keywords:** teneligliptin, dipeptidyl peptidase-4 (DPP-4) inhibitor, type 2 diabetes mellitus (T2DM), systematic review, glycemic control

## Abstract

**Background:** Teneligliptin is a 3rd-generation dipeptidyl peptidase-4 (DPP-4) inhibitor. There is a limited evidence regarding the effect of teneligliptin. Therefore, this study is to assess the efficacy and safety of teneligliptin in type 2 diabetes mellitus (T2DM) patients with inadequately glycemic controlled.

**Methods:** A search of PubMed, Medline, Embase, and The Cochrane Library during 2000.01–2018.03 was performed for randomized controlled trials of teneligliptin compared to placebo in patients with T2DM with monotherapy or add-on treatment.

**Results:** Ten trials with 2119 patients were analyzed. Teneligliptin produced absolute reductions in glycated hemoglobin A1c (HbA1c) levels (weighted mean difference (WMD) 0.82%, 95% confidence interval (CI) [−0.91 to −0.72], *p* < 0.00001) compared with placebo. However, after 36–42 weeks of follow-up (open-label), HbA1c level rise higher than duration (double-blind) in teneligliptin group. Teneligliptin led to greater decrease of fasting plasma glucose (FPG) level (vs. placebo, WMD −18.32%, 95% CI [−21.05 to −15.60], *p* < 0.00001). Teneligliptin also significantly decreased the 2 h post-prandial plasma glucose (2 h PPG) (WMD −46.94%, 95% CI [−51.58 to −42.30], *p* < 0.00001) and area under the glucose plasma concentration-time curve from 0 to 2 h (AUC_0−2h_) for PPG (WMD −71.50%, 95% CI [−78.09 to −64.91], *p* < 0.00001) compared with placebo. Patients treated with teneligliptin achieved increased homeostasis model assessment of β cell function (HOMA-β) with 9.31 (WMD, 95% CI [7.78–10.85], *p* < 0.00001). However, there was no significant difference between teneligliptin and placebo in overall adverse effects (0.96 risk ratio (RR), 95% CI [0.87, 1.06], *p* = 0.06). The risks of hypoglycemia were not significantly different between teneligliptin and placebo (1.16 RR, 95% CI [0.59, 2.26], *p* = 0.66).

**Conclusions:** Teneligliptin improved blood glucose levels and β-cells function with low risk of hypoglycemia in patients with T2DM. Common adverse effects of teneligliptin including hypoglycemia were identified and reviewed. Risks of cardiovascular events are less certain, and more data for long-term effects are needed.

## Introduction

Approximately 425 million adults (one in eleven) are living with diabetes and one in two adults remains undiagnosed worldwide (2017)^1^. Type 2 diabetes mellitus (T2DM) accounts for more than 90% cases of diabetes. T2DM induces microvascular and macrovascular complications, which place a huge burden on patients, caregivers, and health care systems (Chatterjee et al., 2017). A recent statement of Standards of Medical Care in Diabetes by the American Diabetes Association (ADA) has recommended that initial treatment with metformin as monotherapy after inadequate life style modification, followed by sulfonylurea, thiazolidinedione, dipeptidyl peptidase-4 (DPP-4) inhibitor, sodium-glucose cotransporter 2 inhibitor (SGLT2-i), glucagon-like peptide 1 (GLP-1) receptor agonist and insulin alone or in combination (American Diabetes Association, 2017). However, it is still difficult to find an antihyperglycemic agent with long-term glucose control, minimal hypoglycemia, no weight gain and a relatively affordable price (Liao, 2011). DPP-4 inhibitors have been considered as a cornerstone in the management of T2DM because of their robust efficacy and favorable tolerability profiles (Deacon, 2011).

A large body of randomized controlled trials (RCTs) showed that the DPP4 inhibitors such as sitagliptin and vildagliptin were effective for blood glycemic control by declining the HbA1c, FPG, and PPG and improving the function of pancreatic α and β cells. In addition to targeting glycemic control, DPP4 inhibitors have low risk of hypoglycaemia with neutral effect of body weight with a favorable safety profile (Inzucchi et al., 2012; Garber et al., 2015). Clinical trials showed that the relative common adverse events of DPP4 inhibitors are gastrointestinal symptoms, nasopharyngitis, upper respiratory infections and headache in clinical trials. Other less common adverse event such as skin- and immune-related effects are reported in post-marketing surveillance (Filippatos et al., 2014).

Teneligliptin, a 3rd-generation DPP-4 inhibitor, acts as a competitive reversible inhibitor of DPP-4 and decreases the degradation of incretins, especially GLP-1, consequently stimulating insulin secretion and suppressing glucagon secretion in a glucose-dependent manner (Gallwitz, 2010). More importantly, teneligliptin is effective and safe for patients with T2DM with renal impairment, or even end-stage renal disease, without dose adjustments (Abubaker et al., 2017). Teneligliptin was synthesized in Japan and is available in Japan, Argentina, Korea and India. It is currently in phase I clinical trials in the USA and phase II clinical trials in Europe for management of T2DM (Kishimoto, 2013; Scott, 2015). A few clinical studies showed that teneligliptin significantly improves glycemic control, is well tolerated, and causes a low incidence of hypoglycemia when used as monotherapy or combination therapy (Eto et al., 2012; Kadowaki and Kondo, 2013a,b,c; Kim et al., 2015; Bryson et al., 2016; Hong et al., 2016; Kadowaki et al., 2017a). However, there were only few reports of comprehensive profiles of the benefits and risks of teneligliptin in patients with T2DM to date. Thus, we conducted a systematic review and meta-analysis to assess the efficacy and safety of teneligliptin in the management of T2DM either as monotherapy or add-on treatment. In adults, teneligliptin is primarily metabolized by cytochrome P450 (CYP) 3A4 and flavin monooxygenases (FMO) (Patel et al., 2016). Approximately 34% of each administered dose of teneligliptin is excreted unchanged via the renal route, while 66% is metabolized and eliminated via the hepatic and renal routes (Sharma et al., 2016). Teneligliptin is usually orally administered at 20 mg once daily and increased to 40 mg once daily if the dosage is insufficient (Kishimoto, 2013). Thus, we evaluated 20 mg of teneligliptin once daily in the treatment of T2DM patients.

## Materials and methods

### Search strategy and study selection

According to the preferred reporting items for systematic reviews and meta-analyses (PRISMA) statement (Liberati et al., 2009), we systematically searched PubMed, Medline, Embase and the Cochrane Library from inception to March 2018 without a language restriction. Clinical Trials (http://www.clinicaltrials.gov) were also searched. We used the terms “Teneligliptin,” “MP-513,” “T2DM,” and “Type 2 diabetes mellitus” for the searches, and the terms were adjusted to conform to the relevant rules in each database. We also used (“Diabetes Mellitus, Type 2”[Mesh] AND teneligliptin) to search the data. The PROSPERO registration number is CRD42018091232.

Titles and abstracts of all retrieved citations were screened by two independent reviewers (L.X.X and H.X.F) to identify all potentially eligible studies. Full texts were retrieved for relevant records. Any resulting discrepancies were resolved by discussion, with involvement of a third reviewer if necessary.

### Inclusion and exclusion criteria

Trials of teneligliptin for the treatment of T2DM according to the WHO diagnostic criteria were included if they met the following criteria: (1) Patients: any ethnic origin and aged over 18. (2) Interventions: any use of teneligliptin as monotherapy or combination therapy, with a duration of intervention of at least 4 weeks. (3) Comparison: placebo or active comparators with or without background therapy. (4) Outcomes: at least one of the following indicators was reported: (a) glycated hemoglobin A1c (HbA1c), (b) fasting plasma glucose (FPG), (c) 2 h post-prandial plasma glucose (2 h PPG), (d) area under the glucose plasma concentration-time curve from 0 to 2 h (AUC_0−2h_) PPG, (e) homeostasis model assessment of β cell function (HOMA-β), homeostasis model assessment of insulin resistance (HOMA-IR), and (f) adverse events (AEs) such as hypoglycemia. (5) Trial design: randomized controlled trials (RCTs) published or searched in Clinical Trials without language restrictions. We excluded the studies of non-randomized trials, case reports, editorials, letters to the editors, and conference abstracts.

### Data extraction and risk of bias assessment

Two reviewers (L.X.X and H.X.F) independently screened the title and abstract and extracted study characteristics, baseline characteristics, and prespecified outcomes of efficacy and safety. They also independently assessed the risk of bias of randomized controlled trials (Higgins and Green, 2011), including random sequence generation, allocation concealment, blinding of participants and personnel, blinding of outcome assessment, incomplete outcome data, selective reporting, and other bias (i.e., design-specific risks of bias, baseline imbalance, blocked randomization in unblinded trials) with the Cochrane Risk of Bias tool. Any discrepancies were resolved by discussion. We mainly focused on the data for patients randomly assigned to teneligliptin 20 mg/day, which is the most common dose used in clinical practice.

### Statistical analysis

All outcomes were pooled using RevMan 5.3. The efficacy was evaluated by weighted mean difference (WMD, indicators changed from baseline), along with 95% confidence intervals (CIs). The safety was assessed by risk ratios (RRs, incidence of AEs or hypoglycemia), along with 95% CIs. Heterogeneity was assessed by the chi-square test and the I^2^ statistic. If I^2^ < 50%, the fixed-effect model with the Mantel-Haenszel method was used; otherwise, the random-effect model was adopted. If the primary outcome data standard deviation (SD) were missing or incomplete, we e-mailed the corresponding authors or the sponsors to obtain them. When necessary, the values of SD were calculated from SE as described in the Cochrane Handbook.

## Results

### Study selection and characteristics

**Figure 1:**
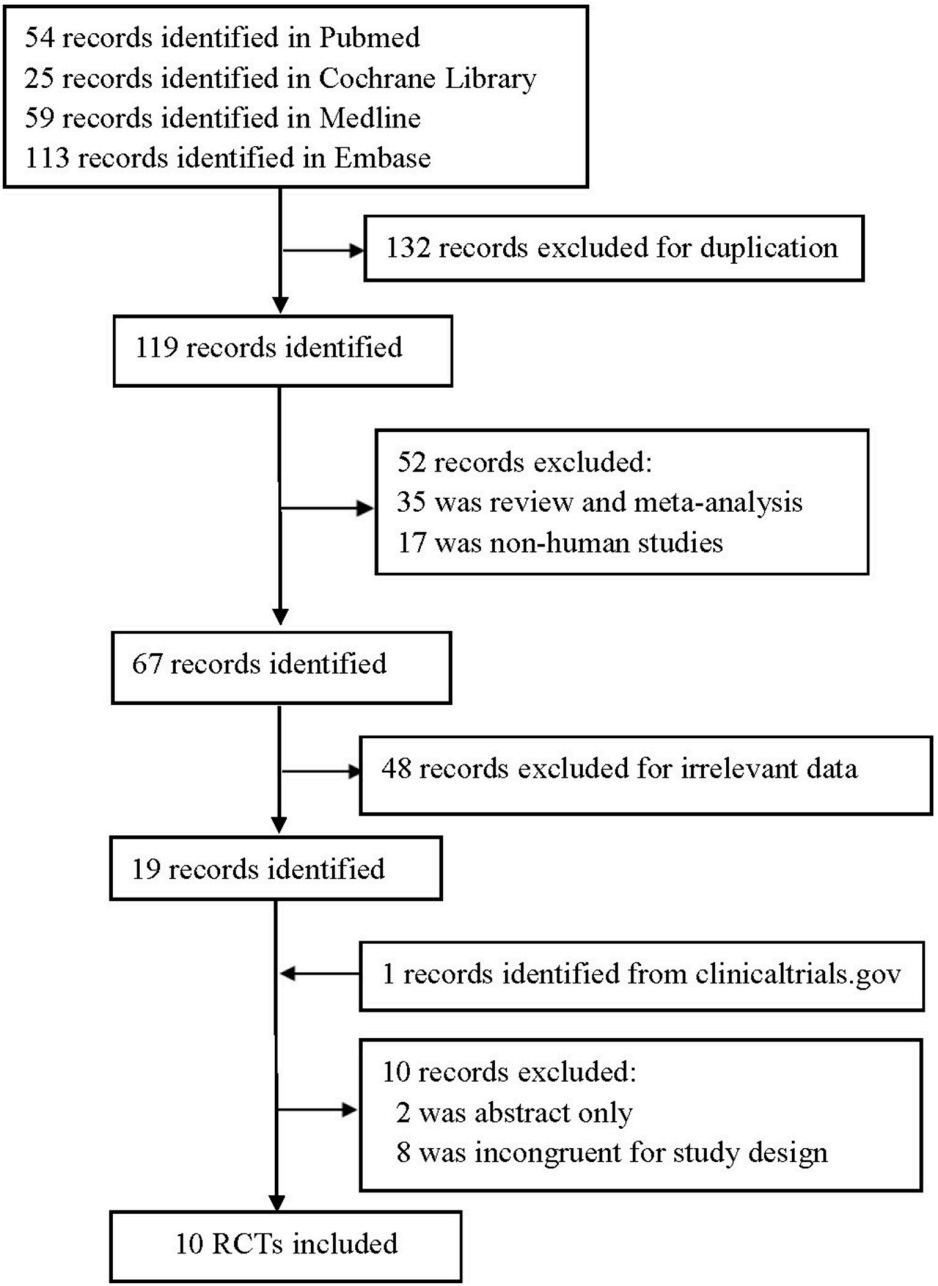
Flow chart of selected study.

We identified 251 publications in four databases, and 132 were excluded for duplication. Nineteen were remained after further removing due to non-human studies, irrelevant data, review or meta-analysis. Finally, a total of 10 RCTs (Eto et al., 2012; Kadowaki and Kondo, 2013a,b,c; Mitsubishi Tanabe Pharma Corporation, 2014; Kim et al., 2015; Bryson et al., 2016; Hong et al., 2016; Kadowaki et al., 2017a,b) (*n* = 2119) met the final inclusion criteria for meta-analysis after excluding 10 and adding one study (Figure 1).

The characteristics of the included RCTs are shown in Table 1. All included trials were double-blind RCTs; two were phase II (Kadowaki and Kondo, 2013c; Bryson et al., 2016), six were phase III (Kadowaki and Kondo, 2013a,b; Mitsubishi Tanabe Pharma Corporation, 2014; Kim et al., 2015; Hong et al., 2016; Kadowaki et al., 2017b), and one was phase IV (Kadowaki et al., 2017a). Trial durations ranged from 4 to 24 weeks. Seven trials had extension periods (ranging from 2 to 42 weeks) (Kadowaki and Kondo, 2013a,b,c; Bryson et al., 2016; Hong et al., 2016; Kadowaki et al., 2017a,b). Mean baseline HbA1c across the study populations ranged from 7.72 to 8.73%; mean baseline FPG ranged from 143.0 to 165.1 mg/dL. Participants in most trials were mainly middle-aged and overweight adults who had T2DM for more than 4 years. Mean age ranged from 55.9 to 60.4 years. Body mass index (BMI) in most trials ranged from 24.8 to 26.5 kg/m^2^.

**Table 1:** Characteristics of randomized controlled trials.

**Study, Year**	**Participants**	**Internentions**	**Age (years)**	**BMI (kg/m2)**	**HbA1c (%)**	**FPG (mg/dL)**	**Body weight (kg)**	**T2DM duration (years)**	**Background therapy**	**Phase**	**Duration (extension)**
Kadowaki et al., 2017a	N: 154	Teneligliptin(20 mg)Placebo	55.9 ± 8.3 54.1 ± 10.2	25.53 ± 3.95 26.50 ± 4.82	7.98 ± 0.80 8.09 ± 0.85	148.5 ± 21.2 151.5 ± 25.3	72.32 ± 12.08 73.58 ± 15.75	8.15 ± 5.86 7.34 ± 5.34	Add on to canagliflozin	III	24 w (2 w)
Kadowaki et al., 2017b	N: 148	Teneligliptin(20 mg)Placebo	60.1 ± 11.3 57.4 ± 12	24.87 ± 3.14 25.11 ± 3.66	8.70 ± 0.81 8.73 ± 0.81	16.9 ± 40.4 162.8 ± 45.0	67.62 ± 12.98 68.61 ± 13.68	11.85 ± 8.14 12.97 ± 9.22	Add on to insulin	IV	16 w (36 w)
Bryson et al., 2016	N: 447	Teneligliptin(20 mg)Placebo	58.3 ± 9.5 58.9 ± 8.2	NR	NR	NR	NR	NR	Add on to metformin	II	24 w (28 w)
Hong et al., 2016	N: 142	Teneligliptin(20 mg)Placebo	56.64 ± 10.07 57.93 ± 11.90	24.96 ± 2.51 25.07 ± 3.23	7.74 ± 0.61 7.74 ± 0.53	155.96 ± 26.14 162.09 ± 31.3	65.81 ± 11.39 66.61 ± 12.15	4.59 ± 3.87 4.59 ± 3.94	Monotherapy	III	24 w (2 w)
Kim et al., 2015	N: 204	Teneligliptin(20 mg) Placebo	55.7 ± 8.7 56.4 ± 9.2	NR	7.79 ± 0.80 7.72 ± 0.65	151.32 ± 35.55 151.31 ± 25.86	NR	6.7 ± 4.8 8.0 ± 5.9	Add on to metformin	III	16 w
NCT00998881 2014	N: 203	Teneligliptin(20 mg)Placebo	58.5 ± 10.0 59.2 ± 10.1	NR	NR	NR	NR	NR	Monotherapy	III	12 w
Kadowaki and Kondo, 2013a	N:324	Teneligliptin(20 mg)Placebo	59.2 ± 9.5 58.5 ± 9.6	24.9 ± 3.9 25.2 ± 3.9	7.8 ± 0.7 8.0 ± 0.7	143.0 ± 26.8 150.0 ± 30.3	NR	6.3 ± 6.4 5.8 ± 5.0	Monotherapy	II	12 w (2 w)
Kadowaki and Kondo, 2013b	N: 204	Teneligliptin(20 mg)Placebo	59.7 ± 9.7 61.1 ± 8.9	26.2 ± 5.2 25.6 ± 3.7	8.1 ± 0.9 7.9 ± 0.8	150.7 ± 28.1 145.7 ± 26.5	70.0 ± 16.6 67.7 ± 13.0	7.2 ± 4.8 7.7 ± 6.1	Add on to pioglitazone	III	12 w (42 w)
Kadowaki and Kondo, 2013c	N: 194	Teneligliptin(20 mg)Placebo	58.4 ± 8.6 60.3 ± 7.8	24.9 ± 3.6 24.6 ± 3.6	8.4 ± 0.8 8.4 ± 0.8	165.1 ± 24.5 163.4 ± 31.3	66.2 ± 12.1 65.9 ± 11.8	9.3 ± 6.7 8.3 ± 6.2	Add on to glimepiride	III	12 w (42 w)
Eto et al., 2012	N: 99	Teneligliptin(20 mg)Placebo	57.1 ± 8.7 58.6 ± 8.9	24.8 ± 3.8 25.7 ± 4.5	8.3 ± 0.8 8.2 ± 1.1	163.1 ± 30.8 153.6 ± 31.9	NR	6.4 ± 5.9 7.8 ± 6.2	Monotherapy	NR	24 w

*BMI, body mass index; HbA1c, glycated hemoglobin; FPG, fasting plasma glucose; T2DM, type 2 diabetes mellitus; NR not report NCT00998881: This study have not been published. Inclusion criteria: patients in the study must have an HbA1c level between 6.5 and 10.0%*.

Teneligliptin was administered before a standard meal at a dose of 20 mg once daily and the efficacy and safety were compared to placebo in all trails. Four trials were monotherapy and six add-on treatments, and the background therapies were canagliflozin, insulin, metformin, pioglitazone, and glimepiride.

### Risk of bias

**Figure 2:**
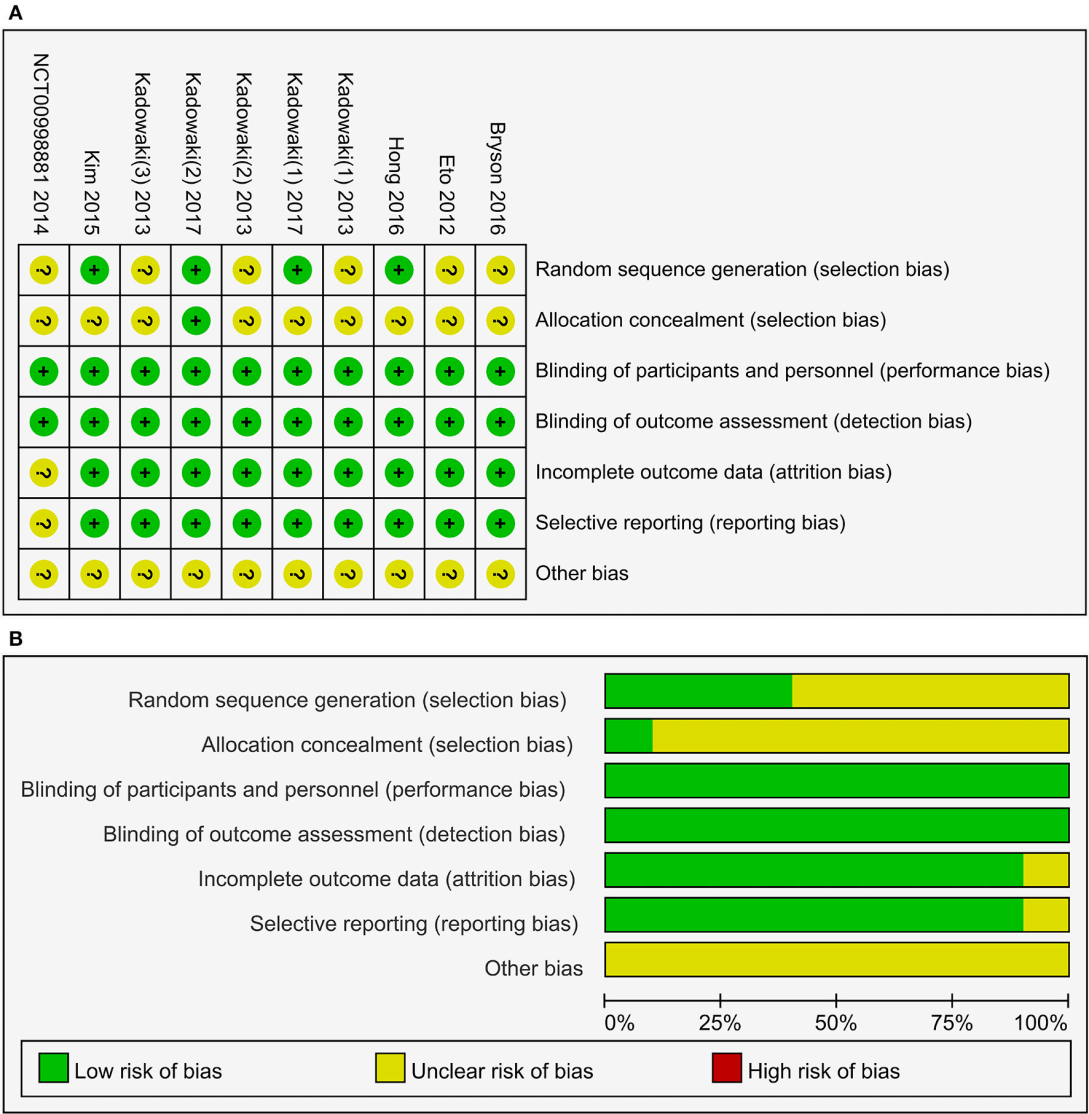
Summary **(A)** and graph **(B)** of the risk of bias in the included trials by Cochrane risk of bias toll based upon reviewers' judgment of each domain.

All the included studies were randomized, double-blind, placebo-controlled studies and had a low risk for bias, as evaluated by The Cochrane Collaboration's tool for assessing risk of bias (Figure 2). However, only four of ten studies elaborated the generation of random sequences and only one record of the allocation concealment.

### HbA1c

**Figure 3:**
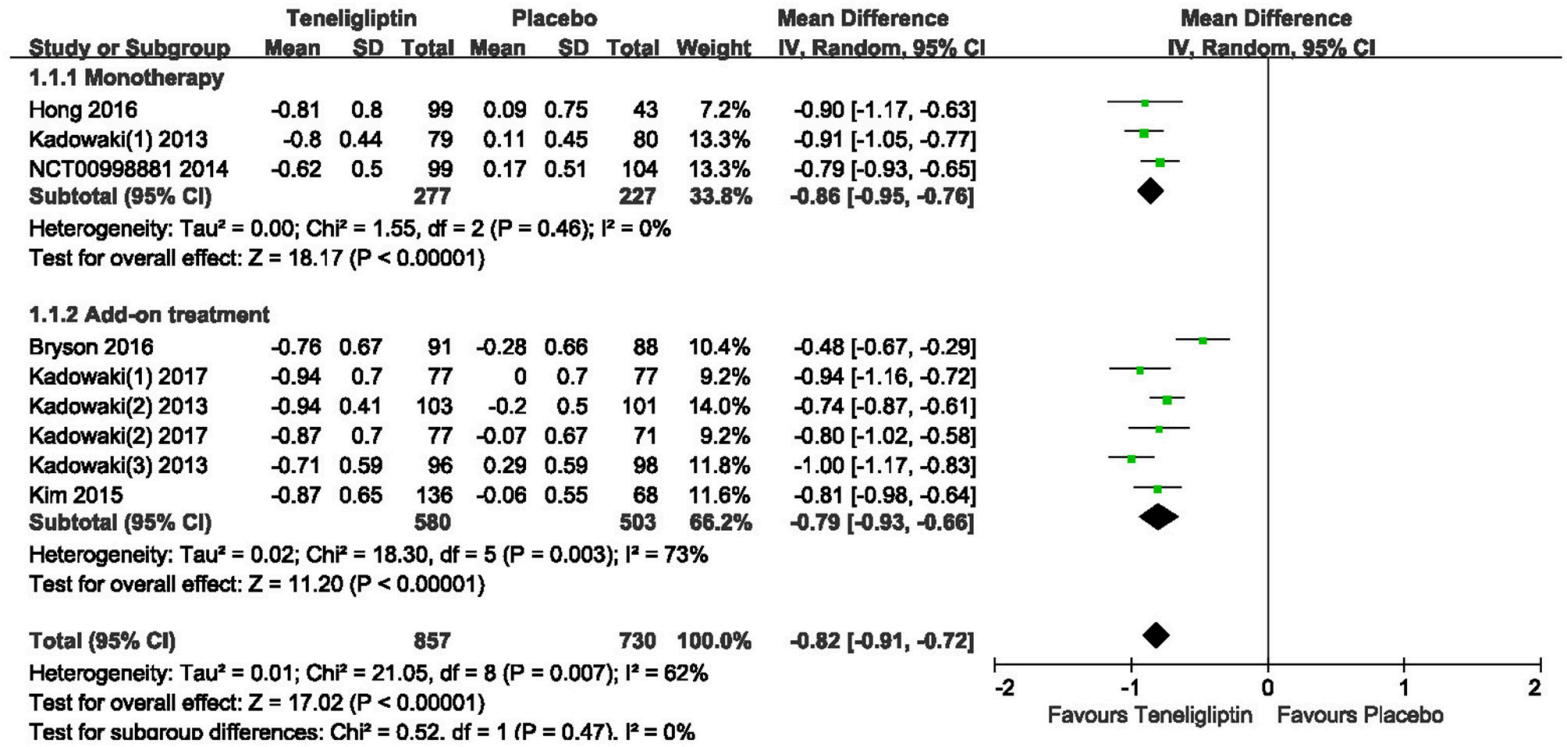
HbA1c change of teneligliptin vs. placebo from the baseline by meta-analysis.

**Figure 4:**
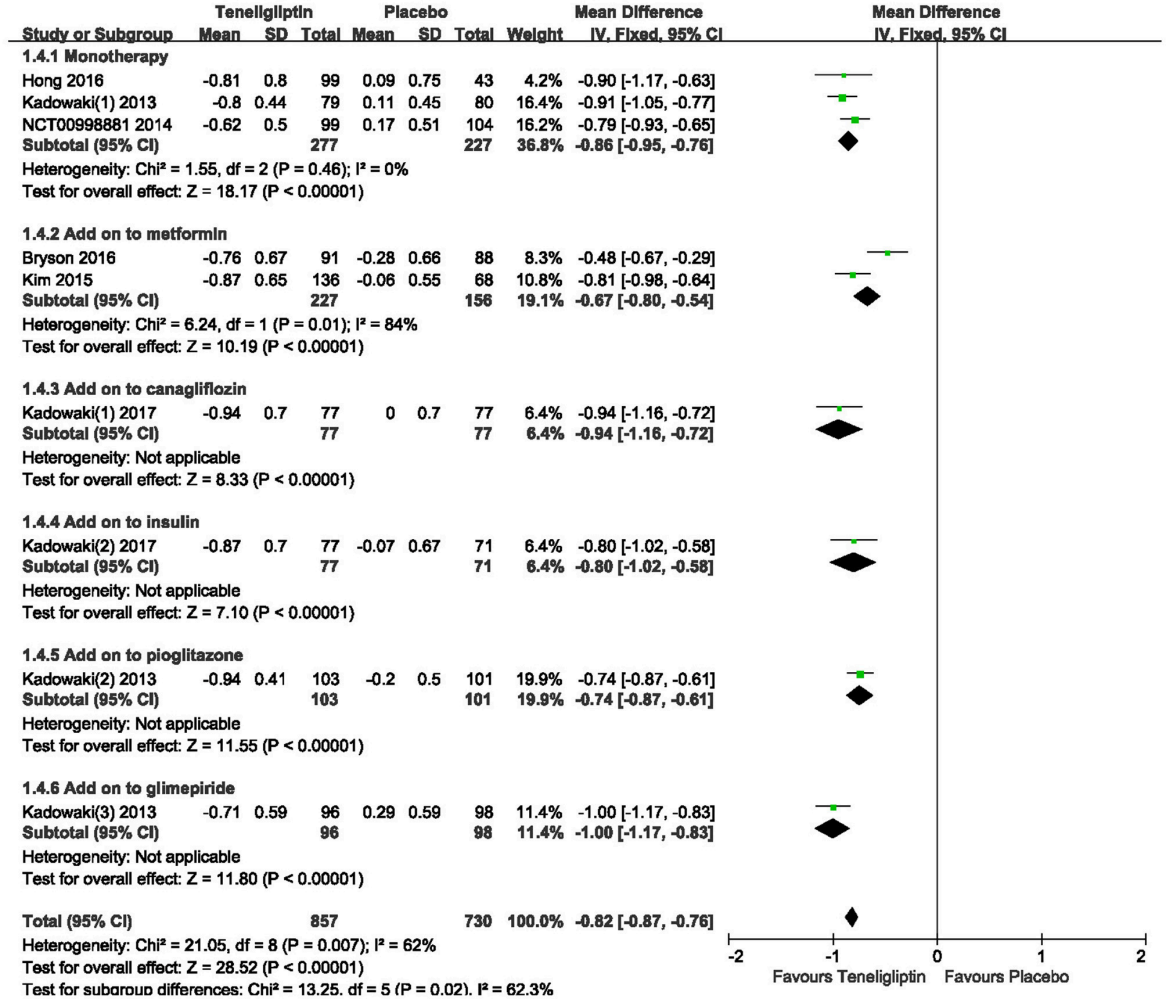
Effect of teneligliptin on HbA1c with different background therapy compared to placebo.

**Figure 5:**
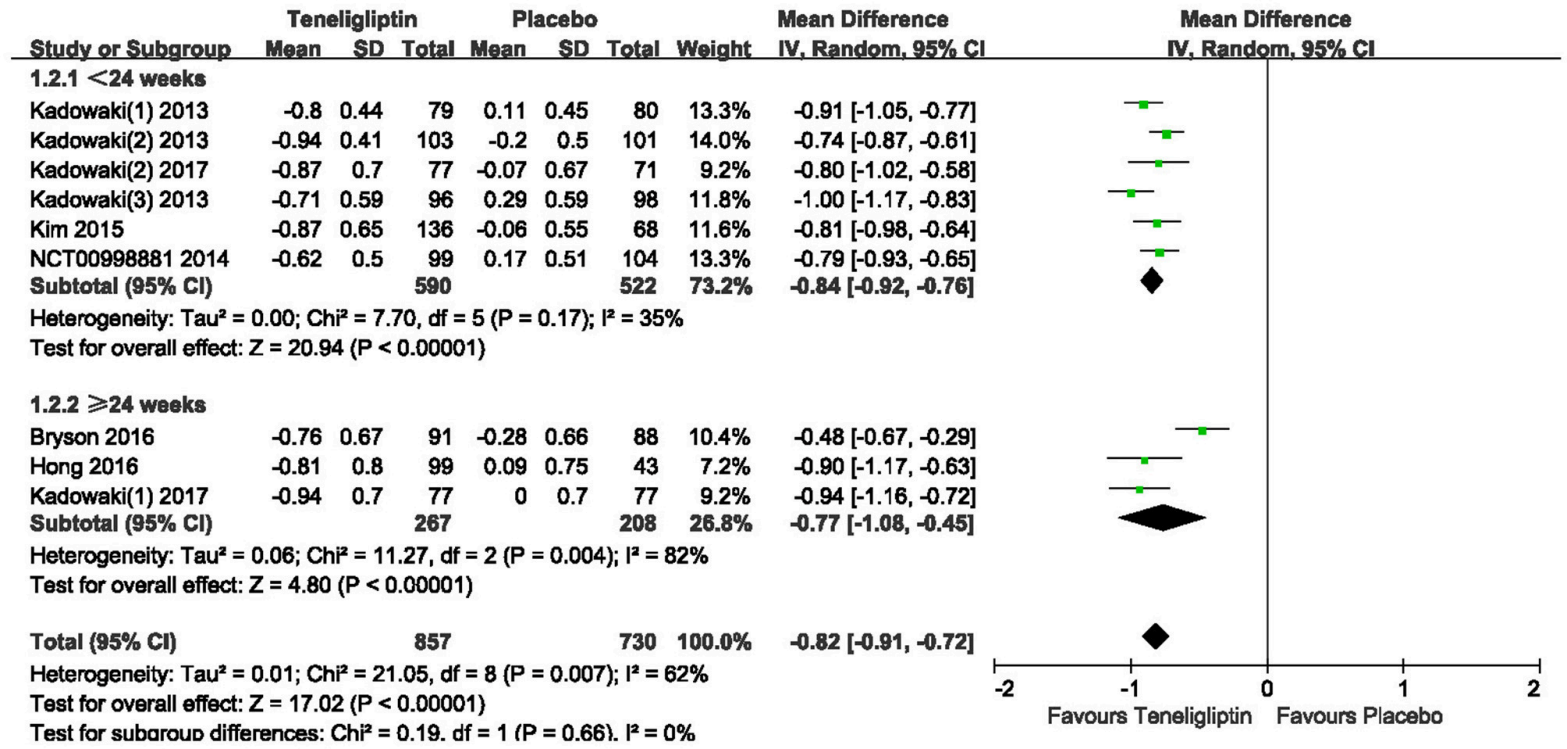
Effect of teneligliptin on HbA1c with different duration of treatment compared to placebo.

**Figure 6:**
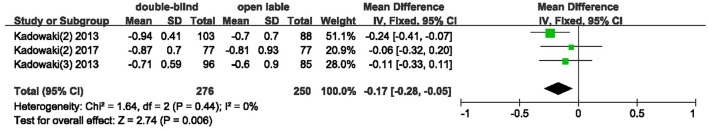
Comparative effect of teneligliptin in double-blind period vs. different follow-up time.

**Figure 7:**
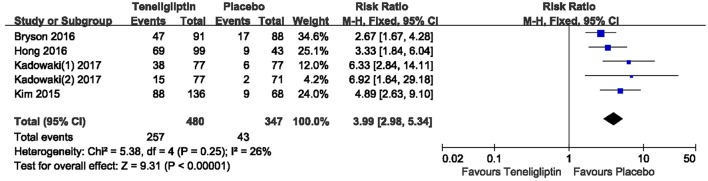
The proportion of patients who achieved HbA1c < 7% treated with teneligliptin vs. placebo by meta-analysis.

The effects of teneligliptin vs. placebo on the HbA1c change from baseline are shown in Figure 3. Teneligliptin significantly reduced HbA1c (WMD −0.82%, 95% CI [−0.91 to −0.72], *p* < 0.00001) as monotherapy (WMD −0.86%, 95% CI [−0.95 to −0.76], *p* < 0.00001), or add-on treatment (WMD −0.79%, 95% CI [−0.93 to −0.66], *p* < 0.00001) compared to placebo. Analyses of Subgroup didn't reduce the high level of heterogeneity with different drugs and different treatment duration (Figures 4, 5). Removing two studies (Kadowaki and Kondo, 2013c; Bryson et al., 2016) because of larger effect size than other trials, the heterogeneity and effect size of HbA1c reduced significantly (−0.82%, −0.89 to −0.76; I^2^ = 0%). 36–42 weeks of follow-up didn't show better decline of HbA1c in teneligliptin group (Figure 6). A greater proportion of subjects received teneligliptin achieved the target of HbA1c < 7% (RR 3.99, 95% CI [2.98–5.34], *p* < 0.00001) compared to placebo (Figure 7).

### FPG

**Figure 8:**
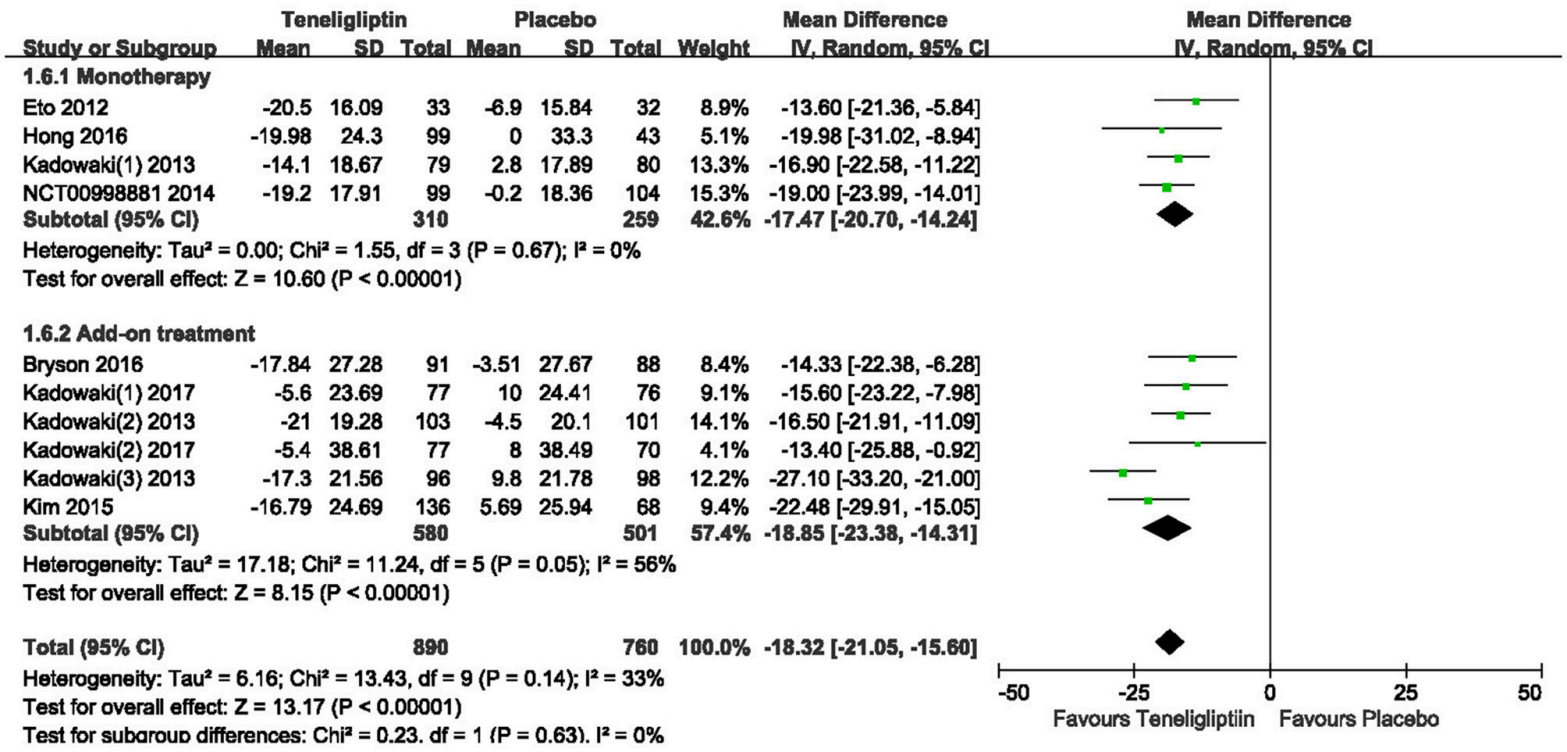
Effects of teneligliptin and placebo on FPG change from the baseline by meta-analysis.

A significant decrease from the baseline in FPG level was also observed in the teneligliptin group compared to placebo (WMD −18.32%, 95% CI [−21.05 to −15.60], *p* < 0.00001) (Figure 8) as monotherapy (WMD −17.47%, 95% CI [−20.70 to −14.24], *p* < 0.00001), or add-on treatment (WMD −18.85%, 95% CI [−23.38 to −14.31], *p* < 0.00001).

### 2 h PPG and AUC_0−2h_ for PPG

**Figure 9:**
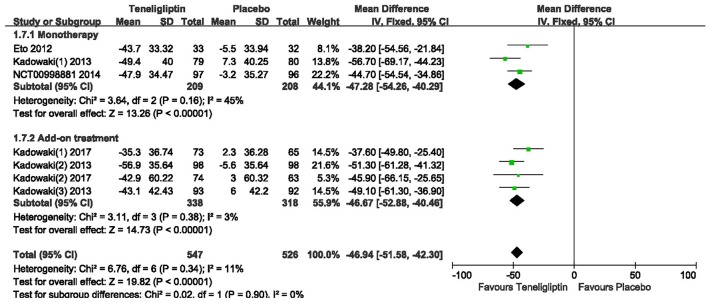
Effects of teneligliptin and placebo on 2 h PPG change from the baseline by meta-analysis.

**Figure 10:**
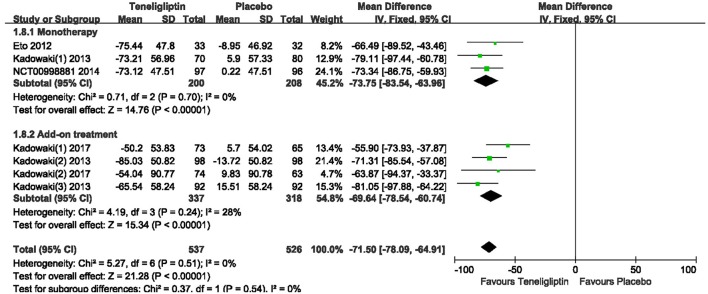
Meta-analysis for Effects of teneligliptin and placebo on AUC_0−2h_ for PPG change from the baseline by meta-analysis.

Teneligliptin significantly decreased the 2 h PPG (WMD −46.94%, 95% CI [−51.58 to −42.30], *p* < 0.00001) and AUC_0−2h_ of PPG (WMD −71.50%, 95% CI [−78.09 to −64.91], *p* < 0.00001) compared to placebo (Figures 9, 10). Teneligliptin reduced 2 h PPG by 47.28 mg/dl as monotherapy (WMD, 95% CI [−54.26 to −40.29], *p* < 0.00001) and by −46.67 mg/dl as combination therapy (WMD, 95% CI [−52.88 to −40.46], *p* < 0.00001). Similarly, the AUC_0−2h_ of PPG was diminished by 73.75 mg·h/dl in the teneligliptin group compared to placebo as monotherapy (WMD, 95% CI [−83.54 to −63.96], *p* < 0.00001) and by 69.64 mg·h/dl as add-on (WMD, 95% CI [−78.54 to −60.74], *p* < 0.00001).

### HOMA-β and HOMA-IR

**Figure 11:**
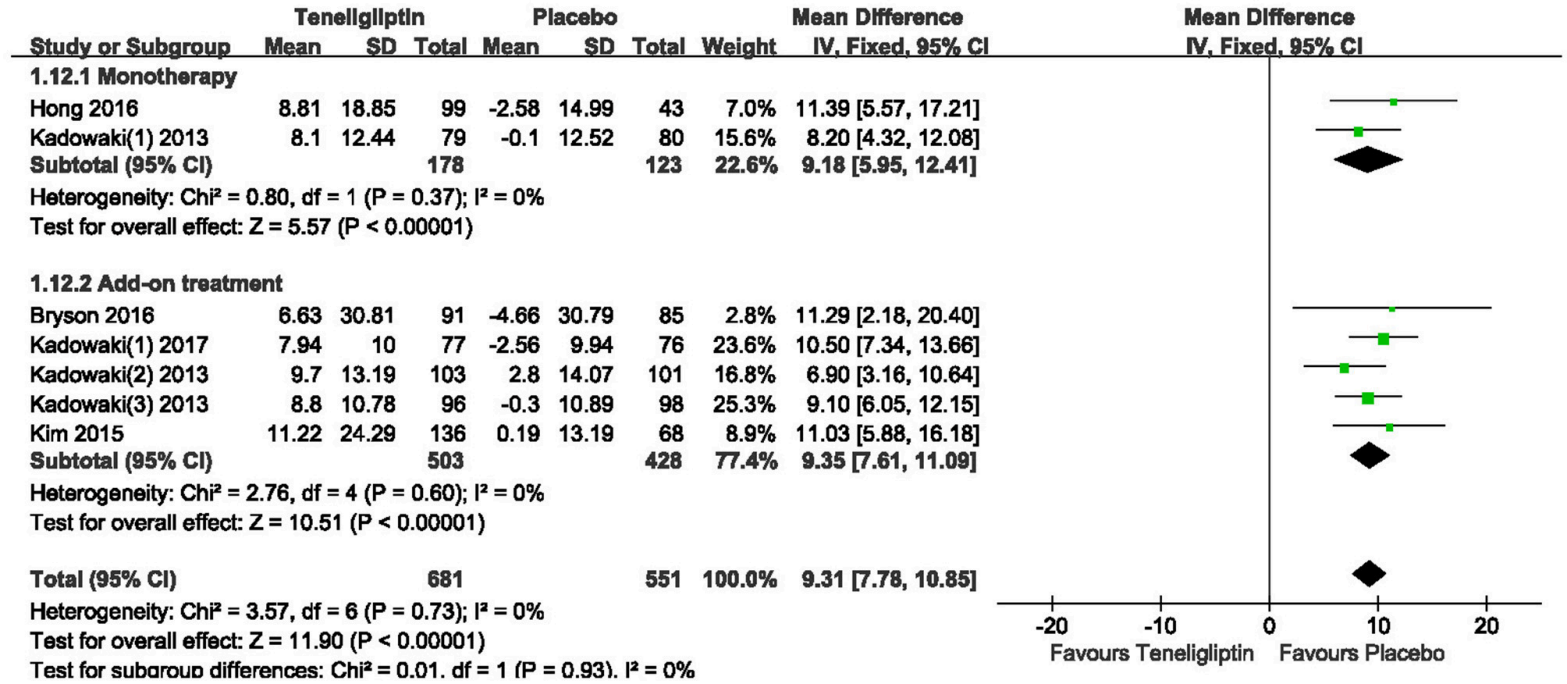
Effects of teneligliptin and placebo on HOMA-β change from the baseline by meta-analysis.

**Figure 12:**
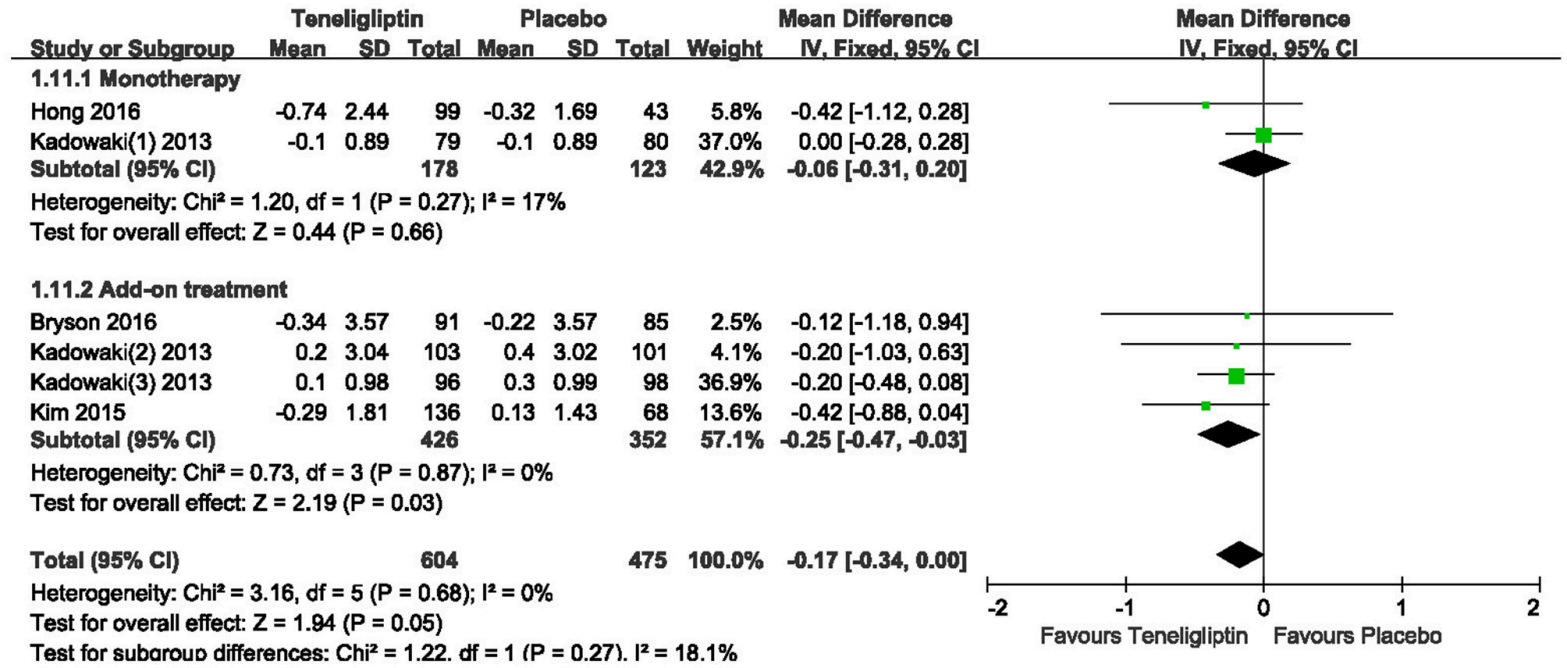
Effects of teneligliptin and placebo on HOMA-IR change from the baseline by meta-analysis.

Patients treated with teneligliptin exhibited increased HOMA-β by 9.31 when all regimens were included (WMD, 95% CI [7.78–10.85], *p* < 0.00001), 9.18 when teneligliptin was used as monotherapy (WMD, 95% CI [5.95–12.41], *p* < 0.00001) and 9.35 when used as add-on treatment (WMD, 95% CI [7.61–11.09], *p* < 0.00001) (Figure 11). Only combined treatment showed statistical significance, with a decrease in HOMA-IR by −0.25 (WMD, 95% CI [−0.47 to −0.03], *p* = 0.03) (Figure 12).

### Overall AEs and hypoglycemia

**Figure 13:**
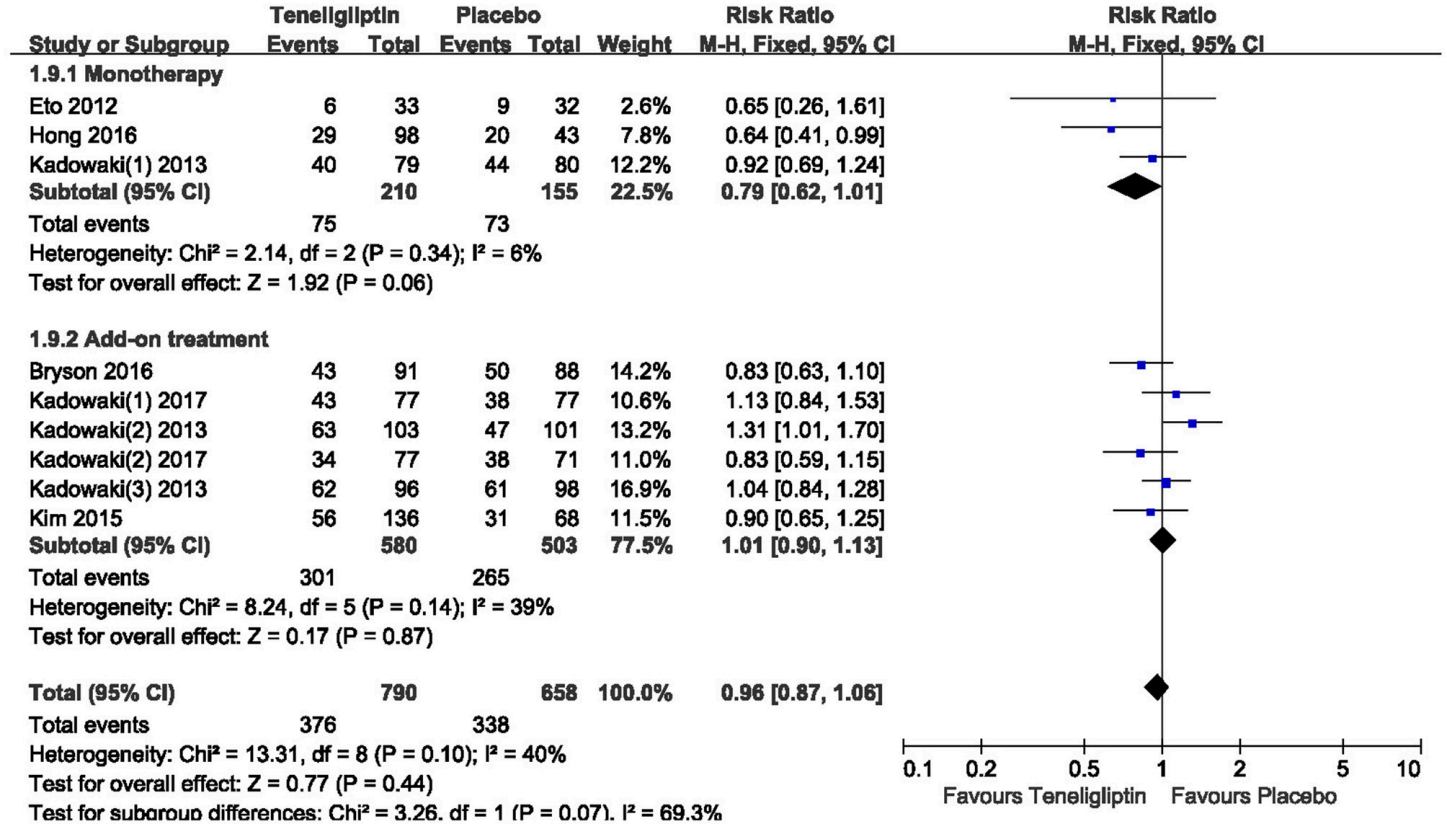
The incidence of AEs of teneligliptin vs. placebo by meta-analysis.

**Figure 14:**
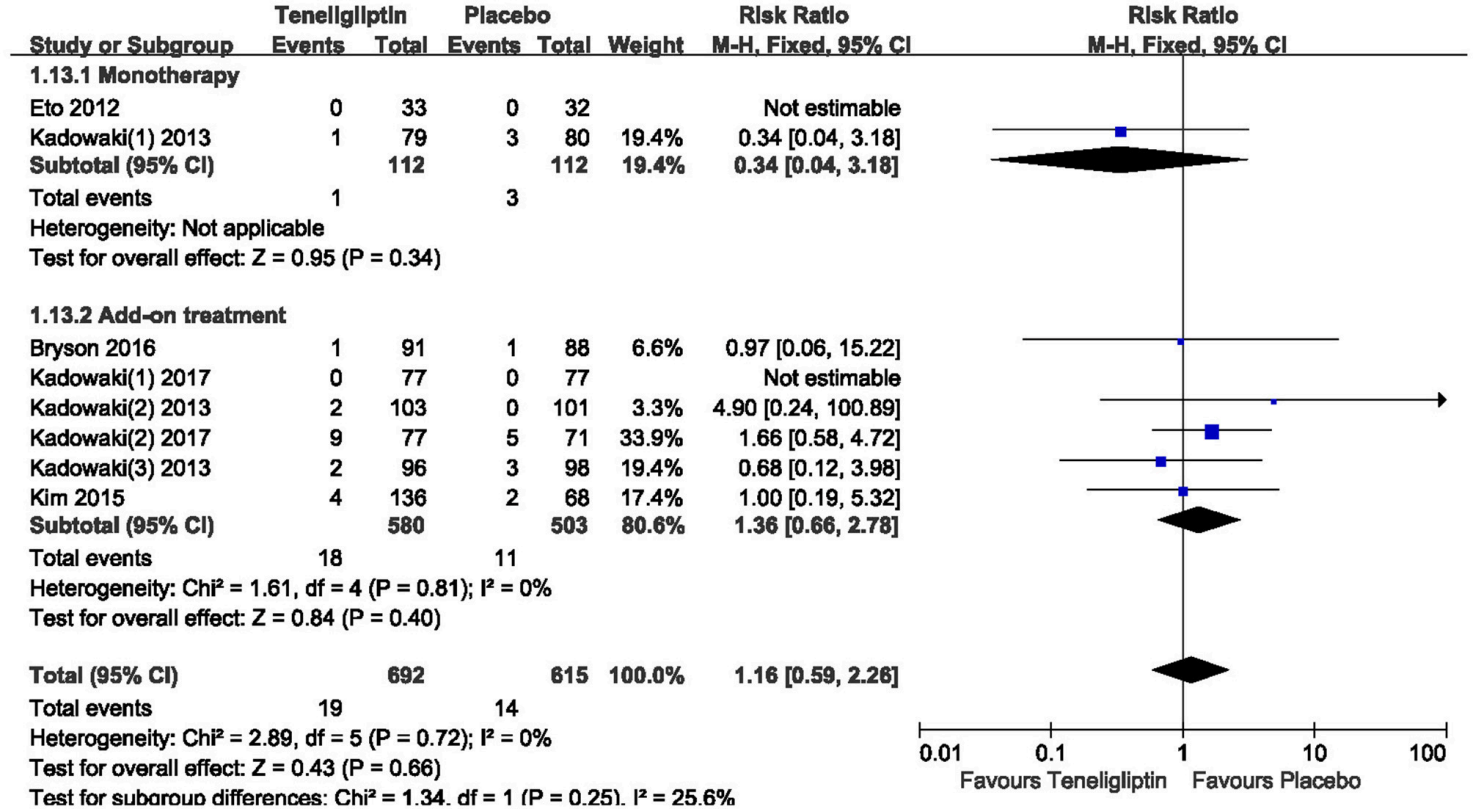
The incidence of hypoglycemia in patients treated with teneligliptin vs. placebo by meta-analysis.

There was no significant difference between teneligliptin and placebo in overall AEs (*p* > 0.05; Figure 13). The incidence of hypoglycemia was low in all included patients, and there was no severe hypoglycemia in most studies. The risk of hypoglycemia was similar between teneligliptin and placebo: 0.34 (RR, 95% CI [0.04,3.18], *p* = 0.34) in monotherapy and 1.36 (RR, 95% CI [0.66, 2.78], *p* = 0.40) in add-on treatment (Figure 14).

## Discussion

According to this meta-analysis, we found that teneligliptin was effective and safe in the treatment of T2DM. HbA1c level is a primary goal for glycemic control (Koren and Rapoport, 2014). Patients treated with teneligliptin had a greater decrease in HbA1c levels from baseline than the placebo group when teneligliptin was used as monotherapy or add-on therapy to insulin or other antidiabetic drugs. Although we performed subgroup analyses, we found a high level of heterogeneity. We speculate that there are two reasons for the high level of heterogeneity. On one hand, accumulating evidence from clinical trials showed that incretin-based drugs were more effective in Asians due to the diverse pathophysiology of type 2 diabetes in different ethnic groups. Asian patients with type 2 diabetes are generally characterized by defective early phase insulin secretion, DPP-4 inhibitors can improve impaired insulin secretion and then exert greater effects in HbA1c in patients with type 2 diabetes (Xueying and Jingbo, 2016). On the other hand, gene-lifestyle interplay in patients with type 2 diabetes from the complex interplay of lifestyle factors acts as a backdrop of inherited DNA risk variants (Paul and Jordi, 2018). It is reasonable to consider that an HbA1c range of 5.7–6.4% (39–47 mmol/mol) is a factor of increased risk for diabetes (American Diabetes Association, 2018). Ideally, the best glycemic control for diabetic patient is to decrease the HbA1c level as close to normal level as possible. Nevertheless, the HbA1c value that is commonly advocated as threshold value for satisfactory glycemic control for most patients is 7%. This value, and less commonly a level of 6.5%, appears in guidelines from the United States, Canada, Europe, Australia and many other countries (Koren and Rapoport, 2014). We found that the effects of DPP4 inhibitors on HbA1c were different after 12 weeks of administration. Treatment of Sitagliptin showed an average decrease in HbA1c levels of 0.65% after 12 weeks, 0.84% after 18 weeks, 0.85% after 24 weeks, 1.0% after 30 weeks, and 0.67% after 52 weeks (Dror, 2011). In addition, omarigliptin reduced HbA1c levels after 66 weeks of follow-up, which was lower than 12 weeks (Philip and Steve, 2016). Our meta-analysis with 3 RCTs showed that the level of HbA1c increased after 36–42 weeks of follow-up time in teneligliptin group, and thus more high-quality and high volume samples were needed for reliable conclusion. A significantly larger proportion of patients treated with teneligliptin achieved an HbA1c level <7% compared to placebo, suggesting teneligliptin is effective in the treatment of patients with T2DM. Teneligliptin lowered not only HbA1c but also other parameters of interest, such as FPG, 2 h PPG and AUC_0−2h_ for PPG. These results indicate that teneligliptin improves glycemic control index and suggest it may be a useful treatment option for patients with T2DM who are inadequately glycemic controlled. For T2DM, body weight is an essential index. However, the included studies in this meta-analysis did not have enough data to assess body weight.

β-Cell destruction plays a key role in the pathophysiology of T2DM, and β-cell conservation delays disease progression (Hong et al., 2016). DPP-4 inhibitors are associated with enhanced β-cell function, making them a good therapeutic option in early disease when the patients still maintain sufficient levels of β-cell function (Kutoh et al., 2014). The HOMA model that is a method for assessing β-cell function and insulin resistance (IR) has proved as a robust clinical and epidemiological tool in evaluation of the pathophysiology of diabetes (Wallace et al., 2004). According to this study of meta-analysis, HOMA-β was significantly improved when teneligliptin was used either as monotherapy or add-on treatment. These facts suggest that teneligliptin has a beneficial effect on pancreatic β-cells and could modestly improve pancreatic function. However, no significant difference in HOMA-IR was observed in the present study.

Teneligliptin has a unique structure with five consecutive rings. Thus, teneligliptin acts on the S2 extensive subsite of DPP-4; this interaction enhances its potency and selectivity (Yoshida et al., 2012; Nabeno et al., 2013; Abubaker et al., 2017). Because of its high selectivity, most AEs of teneligliptin were mild and led to few discontinuations in the meta-analysis. The incidences of AEs were not significantly different between the patients treated with teneligliptin and placebo. Although hypoglycemia was the main adverse event for T2DM patients (Sharma et al., 2016), teneligliptin led to a low risk of hypoglycemia. In addition, the cardiovascular effects of DPP-4 inhibitors remain controversial, while one DPP-4 inhibitor (saxagliptin) increased the risk of hospitalization for heart failure in the overall population; another(alogliptin) showed inconsistent effects on heart failure hospitalization across subgroups of patients, and a third(sitagliptin) had no effect on heart failure (Secrest et al., 2017). The effect of teneligliptin on cardiovascular complication remains unclear. Therefore, it is needed for rational design for longer period of dosing and more RCTs from multicenter for more reliable conclusions.

Teneligliptin is still a relatively new drug, and published clinical studies concerning this drug are sparse (Kishimoto, 2013). The present study is the first meta-analysis about teneligliptin, including 10 high qualities of RCTs. However, it still has several potential limitations. First, there were relatively few patients in the 10 RCTs, which limited our ability to reach clear conclusions about the effects and safety of teneligliptin in the treatment of patients with T2DM. The use of GRADE system (Table 2) suggests that the classification is low or very low, and thus the estimates of the effects are insufficiently. Second, the durations and extensions of the 10 RCTs were relative short, and longer follow-up is needed to evaluate the long-term benefits and risks of teneligliptin. Third, only placebo-controlled RCTs were conducted. Trials are need to be conducted to assess the therapeutic effect of teneligliptin by comparing the effects of other active agents. Fourth, we did not assess the 24 h glucose fluctuations. Large fluctuations in glucose levels may increase the risk of complications, such as cardiovascular disease, so it is better to evaluate post-prandial glucose fluctuations over the entire 24 h dosing interval (Tanaka et al., 2014; Morishita and Nakagami, 2015). In addition, there is a lack of cost-effectiveness studies of teneligliptin. More data will be necessary for a better, more comprehensive analysis.

**Table 2:** Quality of included studies, assessed using the GRADE system.

**Quality assessment**							**No of patients**		**Effect**		**Quality**
**No of studies**	**Design**	**Risk of bias**	**Inconsistency**	**Indirectness**	**Imprecision**	**Other considerations**	**Teneligliptin vs. Placebo**	**Control**	**Relative (95% CI)**	**Absolute**	
**HbA1c**											
9	Randomized trials	Not serious	Serious^a^	Serious^b^	Not serious	Reporting bias^c^	857	730	–	MD 0.82 lower (0.91–0.72 lower)	⊕○○○ VERY LOW
**HbA1c<24 weeks**											
6	Randomized trials	Not serious	Not serious	Serious^b^	Not serious	Reporting bias^c^	590	522	–	MD 0.84 lower (0.92–0.76 lower)	⊕⊕○○ LOW
**HbA1c ≥24 weeks**											
3	Randomized trials	Not serious	Not serious	Serious^b^	Serious^d^	Reporting bias^c^	267	208	–	MD 0.77 lower (1.08–0.45 lower)	⊕○○○ VERY LOW
**HbA1c <7%**											
5	Randomized trials	Not serious	Serious^e^	Not serious	Serious^d^	Reporting bias^c^	257/480 (53.5%)	43/347 (12.4%)	RR 3.99 (2.98–5.34)	371 more per 1000 (from 245 more to 538 more)	⊕○○○ VERY LOW
**FPG**											
10	Randomized trials	Not serious	Not serious	Serious^b^	Not serious	Reporting bias^c^	890	760	–	MD 18.32 lower (21.05–15.6 lower)	⊕⊕○○ LOW
**2 h-PPG**											
7	Randomized trials	Not serious	Not serious	Serious^b^	Not serious	Reporting bias^c^	547	526	–	MD 46.94 lower (51.58–42.3 lower)	⊕⊕○○ LOW
**PPG AUC**_0−2h_											
7	Randomized trials	Not serious	Not serious	Serious^b^	Not serious	Reporting bias^c^	537	526	–	MD 71.5 lower (78.09–64.91 lower)	⊕⊕○○ LOW
**HOMA-IR**											
6	Randomized trials	Not serious	Serious^e^	Serious^b^	Not serious	Reporting bias^c^	604	475	–	MD 0.17 lower (0.34 lower to 0 higher)	⊕○○○ VERY LOW
**HOMA-**β											
7	Randomized trials	Not serious	Not serious	Serious^b^	Not serious	Reporting bias^c^	681	551	–	MD 9.31 higher (7.78–10.85 higher)	⊕⊕○○ LOW
**AEs**											
9	Randomized trials	Not serious	Not serious	Not serious	Not serious	Reporting bias^c^	376/790 (47.6%)	338/658 (51.4%)	RR 0.96 (0.87–1.06)	21 fewer per 1,000 (from 67 fewer to 31 more)	⊕⊕⊕○ MODERATE
**Hypoglycemia**											
8	Randomized trials	Not serious	Serious^e^	Not serious	Serious^d^	Reporting bias^c^	19/692 (2.7%)	14/615 (2.3%)	RR 1.16 (0.59–2.26)	4 more per 1,000 (from 9 fewer to 29 more)	⊕○○○ VERY LOW

a *High level of heterogeneity was observed despite performing subgroup analysis*.

b *Surrogate marker*.

c *Publication bias strongly suspected*.

d *Very small sample size*.

e *Large variation in effect*.

## Conclusion

This meta-analysis suggests that treatment of teneligliptin provided clinically and statistically significant reductions in HbA1c and FPG levels in patients with T2DM. These effects were associated with significant improvements in β-cell function. Furthermore, the incidences of AEs were not significantly in patients treated with teneligliptin compared to placebo. Therefore, the present study demonstrated that teneligliptin exhibits beneficial effects in T2DM patients. However, it is warranted to further investigate with more rational, and longer duration of drug administration from multicenter study and ongoing monitoring due to the potential risks of unclear AEs.

## Author contributions

XL, XH, CB, YY, and JW conceived and designed the review; XL, XH, CB, DQ, SC, QM, YY, and JW reviewed the literature; XL, XH, CB, YY, SC, and JW wrote the manuscript.

### Conflict of interest statement

The authors declare that the research was conducted in the absence of any commercial or financial relationships that could be construed as a potential conflict of interest.

## Footnotes

^1^IDF diabetes atlas - 8th edition [EB/OL]. http://www.diabetesatlas.org/

## Abbreviations

T2DM: type 2 diabetes mellitus
DPP-4: dipeptidyl peptidase-4
HbA1c: glycated hemoglobin A1c
WMD: weighted mean difference
CI: confidence interval
RR: risk ratio
FPG: fasting plasma glucose
PPG: post-prandial plasma glucose
HOMA-β: Homeostasis model assessment of β cell function
HOMA-IR: homeostasis model assessment of insulin resistance
AEs: adverse events
RCTs: randomized controlled trials
AUC_0−2h_: area under the glucose plasma concentration-time curve from 0 to 2 h.
